# Supplementary figures and images for: Sex-specific regulation of the cortical transcriptome in response to sleep deprivation
Source: Front Neurosci. 2024 Mar 5;17:1303727. doi: 10.3389/fnins.2023.1303727 (PMC10948409; doi:10.3389/fnins.2023.1303727)

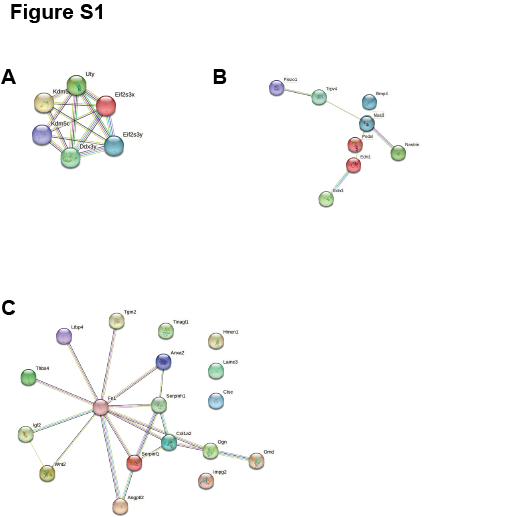

Supplement: Supplementary file 2 [file Image_1.png]

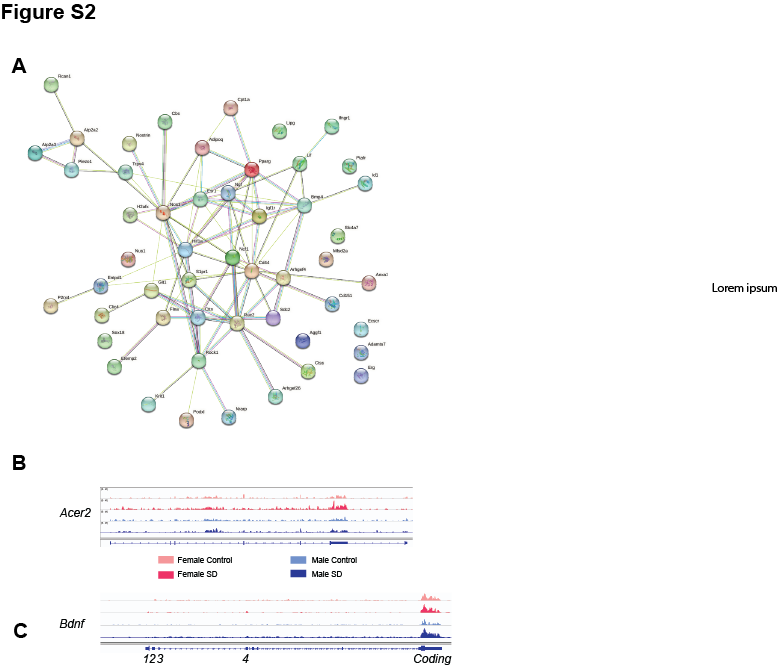

Supplement: Supplementary file 3 [file Image_2.png]
